# Risk factors for transmission of *Salmonella* Typhi in Mahama refugee camp, Rwanda: a matched case-control study

**DOI:** 10.11604/pamj.2018.29.148.12070

**Published:** 2018-03-13

**Authors:** Jose Nyamusore, Marie Rosette Nahimana, Candide Tran Ngoc, Olushayo Olu, Ayodeji Isiaka, Vedaste Ndahindwa, Lakruwan Dassanayake, André Rusanganwa

**Affiliations:** 1 Rwanda Biomedical Center, Kigali, Rwanda; 2 World Health Organization Country Office, Kigali, Rwanda; 3 World Health Organization Country Office, Lagos, Nigeria; 4 School of Public Health, Kigali, Rwanda; 5 UNHCR, Kigali, Rwanda

**Keywords:** Salmonella typhi, typhoid fever, epidemiology, Burundi, refugee camps, risk factors, case-control study

## Abstract

**Introduction:**

In early October 2015, the health facility in Mahama, a refugee camp for Burundians, began to record an increase in the incidence of a disease characterized by fever, chills and abdominal pain. The investigation of the outbreak confirmed *Salmonella* Typhi as the cause. A case-control study was conducted to identify risk factors for the disease.

**Methods:**

A retrospective matched case-control study was conducted between January and February 2016. Data were obtained through a survey of matched cases and controls, based on an epidemiological case definition and environmental assessment. Odd ratios were calculated to determine the risk factors associated with typhoid fever.

**Results:**

Overall, 260 cases and 770 controls were enrolled in the study. Findings from the multivariable logistic regression identified that having a family member who had been infected with S. Typhi in the last 3 months (OR 2.7; p < 0.001), poor awareness of typhoid fever (OR 1.6; p = 0.011), inconsistent hand washing after use of the latrine (OR 1.8; p = 0.003), eating food prepared at home (OR 2.8; p < 0.001) or at community market (OR 11.4; p = 0.005) were risk factors for typhoid fever transmission. Environmental assessments established the local sorghum beer and yoghurt were contaminated with yeast, aerobic flora, coliforms or Staphylococcus.

**Conclusion:**

These findings highlight the need of reinforcement of hygiene promotion, food safety regulations, hygiene education for beverage and food handlers in community market and intensification of environmental interventions to break the transmission of S.Typhi in Mahama.

## Introduction

Typhoid fever is an infectious disease caused by *Salmonella* Typhi [1]. It is transmitted through ingestion of food and water contaminated by the feces and urine of patients, or symptomatic carriers of the bacteria [2]. It is characterized by severe systemic illness with insidious onset, fever, severe headache, malaise, anorexia and relative bradycardia [3]. The clinical scope varies from mild illness with a low-grade fever to severe clinical disease with abdominal discomfort and multiple complications, including intestinal hemorrhages and perforations of the ileum [4]. Recent estimates showed that there are approximately 20.6 million cases and 223,000 typhoid-related deaths annually worldwide [5]. Outbreaks of the disease have been documented in many countries and are associated with poor sanitation, inadequate hygiene practices and unsafe food and drinking water [6]. The political crisis in Burundi, a country located in Eastern Africa, which started in late April 2015, forced thousands of Burundians to flee their homes. This resulted in an influx of refugees into neighboring countries, including Rwanda. In April 2015, a refugee camp was established in the Mahama sector of Kirehe District in the Eastern Province, to accommodate some of these refugees. As of February 2016, the number of refugees living in the camp was estimated to be 48,000 [7]. Commencing in early October 2015, the camp health facility began to record an increase in the incidence of a disease characterized by fever, chills and abdominal pain. The index case was a 24-year-old male, who presented at the Mahama camp health center on 6 October 2015, complaining of fever, chills, headache, abdominal pain, diarrhea and vomiting, lasting for an unknown period of time. He was treated with antibiotics for 7 days without improvement and subsequently referred to the nearby Kirehe district hospital for further investigation, as a suspected case of sepsis. In the following days, several cases with similar symptoms presented at the health center. The Epidemic Surveillance and Response (ESR) Division of the Rwanda Biomedical Center (RBC), the Rwanda School of Public Health, the World Health Organization (WHO) Country Office and the Office of the United Nations High Commissioner for Refugees (UNHCR) conducted a joint epidemiological investigation and environmental assessment of the suspected outbreak from 28 to 29 November 2015. S. Typhi was isolated in 42.1% (n = 8/19) of blood samples and the Ministry of Health (MOH) declared an outbreak of typhoid fever on 1 December 2015 [8]. Following confirmation of the outbreak, prevention and control measures such as active surveillance, case management, community mobilization and hygiene education, improvement in sanitation and provision of clean water were scaled up in the camp. Despite these interventions, which resulted in availability of adequate clean water and sanitation facilities, the number of cases continued to increase, demonstrating the need for a better understanding of the risk factors and pathways for transmission of the disease. The objectives of the study were to characterize further the epidemiology of the outbreak, identify risk factors for transmission and to use these data to propose additional recommendations for controlling S. Typhi outbreaks in Mahama and other refugee camps. The study tested the null hypothesis that "the risk factor for transmission of the outbreak is not consumption of contaminated water"

## Methods

**Study design:** We conducted a retrospective case-control study from January to February 2016, in the Mahama refugee camp, to identify the risk factors associated with the transmission of S. Typhi. Epidemiological data were collected through a survey of matched cases and controls. We also conducted an environmental assessment through collection of environmental samples from community water sources and markets and observation of hygiene practices.

**Study area:** Mahama camp is located in a remote area in Eastern Province, Rwanda, next to the Akagera River, which separates Rwanda from the United Republic of Tanzania. Half of the population is women and 47% of refugees are under 18 years of age [7]. The population lives in 18,481 households, in 15 villages, numbered from 1 to 11 on the old site of the camp (Mahama I) and 17 to 20 on the new site (Mahama II) [7]. Refugees in Mahama I reside in tents, while those in Mahama II live in semi-permanent houses. Water supply to both sites is via a mini-water treatment plant, which draws water supplies from the Akagera River. As of January 2016, water supply to the camp had exceeded the Sphere standard recommendation of 15 liters per person per day [9]. Human waste disposal is through pit latrines, which are strategically located in the camp. Two health centers offer both therapeutic and preventive services, and are sufficiently staffed by qualified doctors, nurses and biotechnologists, however only one was fully functional at the time of this study. Public health challenges in the camp include poor sanitation and hygiene, overcrowding and childhood malnutrition.

**Sample size, methods and exclusion criteria:** For the purpose of this study we used the epidemiological definition, which defined a typhoid fever case as any person who presented with fever for 3 or more days with or without: malaise; headache; abdominal pain; constipation or diarrhea; joint pains; chills or cough, from 1 October 2015 to 28 January 2016 and residing in the camp since its establishment in May 2015. Controls were defined as any person who did not fall into the epidemiological case definition but resided in the camp. The exclusion criteria for cases were any person who was not a permanent resident of Mahama camp. Exclusion criteria for the controls was anyone who had been permanent resident for less than 1 month and anybody who had developed symptoms similar to those of typhoid fever in the past 6 months. We calculated the sample size using a method described by Lemeshow et al [10]. This method established a sample size of 259 cases; based on a ratio of one case to three controls, the number of controls was calculated to be 777, giving a total sample size of 1,036. Cases were selected using a systematic random sampling technique from a database of all cases. The data collection team worked with community leaders to identify and visit the households of the cases. For every typhoid fever case (suspected or confirmed), participants enrolled on the study and three neighborhood controls, matched for age and gender, were identified and enrolled. Each control was selected using a systematic selection method in which the interviewer, after exiting the household of the case, chose the first matched control from the second household on the right side of the case house then skipped two households before choosing the second matched control; the procedure was repeated on the left side of the case house to identify the third control. In the event that the selected household had no eligible candidate, the surveyor moved to the next household without skipping any houses.

**Epidemiological data collection and analyses:** A structured questionnaire was developed with questions on sociodemographic characteristics, signs, symptoms and potential risk factors for typhoid fever. Interviewers were selected from nurses and laboratory technicians from neighboring Rwandan health facilities. The interviewers were trained and used to pilot the questionnaire. Data were reviewed on a daily basis to ensure appropriate matching of cases and their respective controls and to ensure that all questionnaires were appropriately completed. Survey data were entered into Census and Survey Processing System (CSPro) software (version 6.1), via internet-enabled tablet computers and automatically uploaded to a central database, developed with Stata software (version 13). Descriptive analysis by time, place and person for all cases were included in the descriptive analysis. Bivariate analysis and the multivariable logistic regression were conducted to identify risk factors for transmission. Additionally a clustered logistic regression permitted to adjust standard errors and confidence intervals of potential risk factors at village level. An overall goodness-of-fit was also performed for the multivariable model. Variables with a p-value < 0.05 were included in the multivariable logistic regression. Unadjusted and adjusted odds ratios (OR), their 95% confidence interval (CI) and p-values were calculated. Based on the results of the initial data analysis and environmental assessment, which showed clustering of cases around pit latrines, we collected additional data on the risk of living in close proximity to a latrine.

**Environmental samples:** Water samples were collected at random from the mini-water treatment plant, water reservoirs in the camp, water points and from jerry cans at a household level. Physical, chemical and bacteriological analysis of the water samples was performed at the Rwanda Water and Sanitation Corporation laboratory in Kigali. Food and beverages from the community market, restaurants and hawkers within the camp were also sampled and tested for coliforms at the Laboratory for Analysis of Foods, Medicines, Water and Toxics in Kigali.

**Ethical approval and consent to participate:** This study was conducted as part of the extended epidemiological investigation of the typhoid fever outbreak. To ensure confidentiality, no individually identifiable information was collected and the names of interviewees were not displayed in the questionnaire. Rwanda National Ethical Committee provided ethical approval for the study, while administrative clearance was provided by MIDIMAR and UNHCR. Informed consent was obtained from all study participants; respondents over 21 years, and caregivers of participants under 15 years signed the consent form and written agreement was requested from all eligible participants aged between 15 and 20 years. Original agreement and consent forms were in English; however they were translated into Kinyarwanda, the language spoken most widely in Rwanda. Furthermore, approval to conduct the study and publish its result was sought and obtained from WHO (e-Pub number ePub-IP-00043416-EC).

## Results

**Figure 1:**
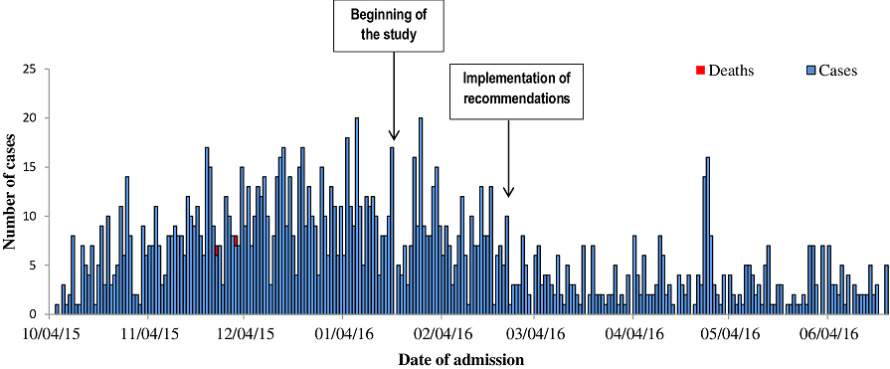
Epidemic curve of typhoid fever in Mahama refugee camp, from October 2015 to June 2016 (n = 1553)

**Descriptive epidemiology of the outbreak:** The analysis of retrospective data collected from patients' clinical records showed that the index case reported to the health facility on 6 October 2015. As of 14 February 2016, 1894 suspected cases were reported and the overall attack rate was 4.0%. Overall, 53.4% (n = 1012) of cases were female, while 46.6% (n = 882) were male. Children under 5 years of age represented 13.6% (n = 257) of the total number of cases and those aged 5-14 years were the most affected, with a proportion of 27.2% (n = 516). The mean age was 20.7 ± 15.8 years and median was 18 years. Of the 15 villages in the study area, village 5 had the highest number of cases (n = 405, 21.4%); while villages 10 and 11 were the most affected, with the highest attack rates of 10.9% and 10.4%, respectively. The peaks displayed on the epidemic curve were separated by an average period of 4-7 days, which falls within the incubation period of typhoid fever, which is 6-30 days, depending on the infective dose [11] (Figure 1).

**Descriptive epidemiology of typhoid cases:** In total, 260 cases and 780 controls were enrolled on the study, however 10 controls were excluded from the final analysis because of data quality issues, giving a total of 770 controls and an overall study population of 1030. Detailed demographic data are presented in Table 1. Overall, 43.6% (n = 449/1030) were male and 56.4% (n = 581/1030) were female. In total, 76.9% (n = 200/260) of cases and 97.1% (n = 575/770) of controls had spent more than 6 months in the camp. The majority of respondents were 5-14 years (24.4%; n = 251/1030). The mean and median age of respondents was 23.5 ± 16.3 years and 22 years, respectively (Table1). Overall, 22.9% (n = 236/1030) of participants resided in village 9.

**Table 1:** Socio-demographic characteristics of the typhoid fever cases and controls in Mahama camp, January to February 2016

Variables	Category	Cases (n=260) n (%)	Controls (n=770) n (%)	Total (n=1030) n(%)
Time spent in the camp	0-6 months	60 (23.1)	195 (25.3)	255 (24.8)
	> 6 months	200 (76.9)	575 (74.7)	775 (75.2)
Age (years)	0–4	26 (10.0)	82 (10.6)	108 (10.5)
	5–14	64 (24.6)	187 (24.3)	251 (24.4)
	15–24	61 (23.5)	160 (20.8)	221 (21.5)
	25–34	61 (23.5)	181 (23.5)	242 (23.5)
	35–44	16 (6.2)	74 (9.6)	90 (8.7)
	45–54	14 (5.4)	41 (5.3)	55 (5.3)
	55 and over	18 (6.9)	45 (5.8)	63 (6.1)
Gender	Male	122 (46.9)	327 (42.5)	449 (43.6)
	Female	138 (53.1)	443 (57.5)	581 (56.4)
Level of education	None	77 (29.6)	218 (28.3)	295 (28.6)
	Pre-school	28 (10.8)	58 (7.5)	86 (8.3)
	Primary	114 (43.8)	360 (46.8)	474 (46.0)
	Secondary	40 (15.4)	125 (16.2)	165 (16.0)
	Tertiary	1 (0.4)	9 (1.2)	10 (1.0)
Occupation since arrival at the camp	None	131 (50.4)	384 (49.9)	515 (50.0)
	Farming	23 (8.8)	82 (10.6)	105 (10.2)
	Trading	5 (1.9)	16 (2.1)	21 (2.0)
	Caterer	8 (3.1)	21 (2.7)	29 (2.8)
	Student	77 (29.6)	240 (31.2)	317 (30.8)
	Other	16 (6.2)	27 (3.5)	43 (4.2)
Religion	None	18 (6.9)	63 (8.2)	81 (7.9)
	Catholic	91 (35)	351 (46.6)	442 (42.9)
	Protestant	101 (38.8)	243 (31.6)	344 (33.4)
	Adventist	13 (5.0)	29 (3.8)	42 (4.1)
	Islam	13 (5.0)	41 (5.3)	54 (5.2)
	Other	24 (9.2)	43 (5.6)	67 (6.5)
Marital status	Never married	144 (55.4)	417 (54.2)	561 (54.5)
	Currently in union	90 (34.6)	273 (35.5)	363 (35.2)
	Separated/ Divorced/ Widowed	19(7.3)	64 (8.3)	83 (8.1)
	Other	7(2.7)	16 (2.1)	23 (2.2)

**Figure 2:**
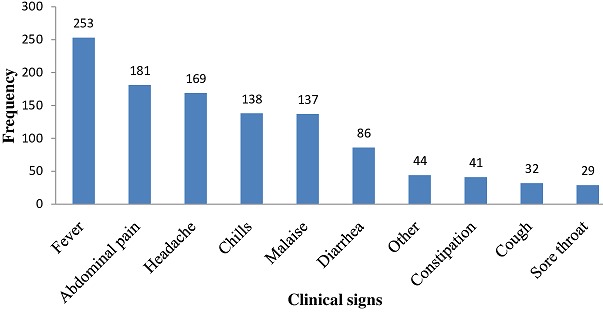
Frequency of clinical symptoms reported by people with typhoid fever in Mahama refugee camp (n = 260)

**Figure 3:**
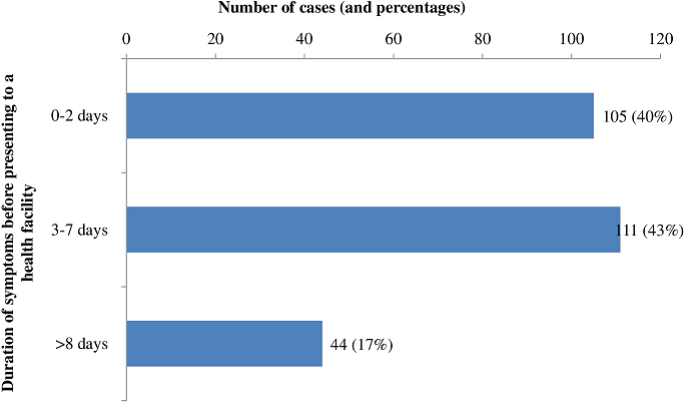
Duration of clinical symptoms prior to presenting to a health facility in Mahama refugee camp, from October 2015 to June 2016

**Clinical signs and symptoms:** The main clinical symptoms were fever (97.3%), abdominal pain (69.6%), headache (65.0%), chills (53.1%) and malaise (52.7%) (Figure 2). Just under half of cases (43.0%) presented to the health facility within 7 days after the onset of the illness (Figure 3). A small proportion (23.5%) of cases self-medicated prior to presenting at health facility. Of those, 28.8% used analgesics and 12.3% used antibiotics. Just over half (59.6%) of patients were admitted to the health facility, where they were treated with intravenous fluids (70.0%) and antibiotics (66.9%) (Table 2).

**Table 2:** Type of treatment received for typhoid fever in Mahama refugee camp, October 2015 to January 2016

Type of treatment	Category	N (%)
Did you receive any treatment at home before going to the health facility?	Yes	61 (23.5)
	No	199 (76.5)
	Total	260 (100.0)
What treatment did you receive before presenting to the health facility?	Antibiotics	32 (12.3)
	Analgesics	75 (28.8)
	Traditional medicines	3 (1.2)
	Don’t know	3 (1.2)
	Other	10 (3.8)
	Total	260 (100.0)
Were you admitted to the health facility?	Yes	155 (59.6)
	No	105 (40.4)
	Total	260 (100.0)
What treatments did you receive at health facility?	IV fluid	182 (70)
	Antibiotics	174 (66.9)
	Don’t know	9 (3.5)
	Other	26 (10.0)
	Total	260 (100.0)

**Bivariate analysis:** The bivariate analysis identified that for people who had spent more than 6 months in the camp, the risk of contracting typhoid fever was nearly three times that of individuals who had spent less than 6 months in the camp (OR 2.89; p < 0.001). Those who completed pre-school education were two times more likely to be infected (OR 2.07; p = 0.021), while participants who had a family member affected by typhoid fever in the past 3 months were more likely to be infected by S. typhi than those whose family members had not been affected by the disease (OR 2.81; p < 0.001). Poor awareness about typhoid fever was also significantly associated with the illness (OR 2.06; p < 0.001); while subjects who reported only sometimes washing their hands after using the latrine were twice as likely to develop typhoid fever compared with those who do it regularly (OR 2.52; p < 0.001). The risk of infection with S. typhi was also higher in respondents who reported eating food prepared at home (OR 4.06; p < 0.001) and those who used the community market as source of food on a daily basis (OR 23.88; p < 0.001), compared with a community kitchen. Respondents who reported only washing their jerry-can sometimes were also more likely to contract typhoid fever (OR 2.07; p = 0.002), compared with those who always washed it (Table 3).

**Table 3:** Risk factors significantly associated with typhoid fever in Mahama refugee camp, 2016

Variables	%	%	UOR (95% CI)	p-value	AOR (95 % CI)	p-value
Overall	**3.9**	**96.1**				
**Time spent in the camp**						
1–6 months	2.0	98	1		1	
More than 6 months	5.6	94.4	2.89 (1.98–4.20)	0.000	1.45(0.89-2.36)	0.134
**Level of completed education**						
None	3.7	96.3	1		1	
Pre-school	7.4	92.6	2.07 (1.11–3.84)	0.021	1.3 (0.68-2.47)	0.43
Primary	3.7	96.3	0.98 (0.66–1.46)	0.925		
Secondary	3.9	96.1	1.04 (0.63–1.72)	0.886		
Tertiary	1.0	99	0.27 (0.03–2.23)	0.221		
**Family members treated for typhoid fever in the past 3 months**						
No	2.9	97.1	1		1	
Yes	7.7	92.3	2.81 (1.95–4.04)	0.000	2.65 (1.84_-_3.79)	0
**Heard about typhoid fever before the outbreak**						
Yes	2.9	97.1	1		1	
No	5.7	94.3	2.06 (1.48–2.86)	0.000	1.63 (1.12–2.38)	0.011
**Washing hands after using the latrine**						
Always or most of the times	3.1	96.9	1		1	
Sometimes or Never	7.5	92.5	2.52 (1.77–3.57)	0.000	1.78 (1.21–2.62)	0.003
**Most common source for food**						
Community kitchen	1.3	98.7	1		1	
Prepared at home	5.0	95 .0	4.06 (2.42–6.80)	0.000	2.75 (1.53–4.96)	0.001
Community market	23.7	76.3	23.88 (4.87–116.95)	0.000	11.39 (2.1061.75)	0.005
**Frequency of jerry-can washing**						
Always	3.3	96.7	1			
Often	4.9	95.1	1.58 (0.98–2.53)	0.058		
Sometimes	6.4	93.6	2.07 (1.32–3.25)	0.002	1.33 (0.80-2.23)	0.265
**Case n=260; Controls n=770**						

**Multivariable logistic regression:** The multivariable regression analysis identified having a family member who had typhoid fever in the last 3 months (OR 2.65; p < 0.001), low awareness about typhoid fever (OR 1.63; p = 0.011) and inconsistent hand washing after use of the latrine (OR 1.78, p = 0.003), were risk factors significantly associated with typhoid fever. Additionally, the risk of developing the disease was higher for respondents who reported eating food prepared at home (OR 2.75; p < 0.001) or food prepared at community market (OR 11.39; p = 0.005) compared with a community kitchen (Table 3). Table 3 shows results from the clustered logistic regression. The goodness-of-fit test showed no significant difference between the clustered and non-clustered multivariate analysis.

**Additional epidemiological analysis:** In total, 64 households ((32 near (less than 15 meters from latrine) and 32 far (25 meters from the latrines)) and 348 people (184 and 164 from households near and far from the latrines, respectively) were surveyed to identify the risk of living in close proximity to a latrine. The proportion of cases in households near and far from the latrines was 16.8% (n = 31/184) and 3% (n = 5/164), respectively; people living near the toilets were 7.7 (p = 0.001) more likely to be affected compared with those living further away.

**Laboratory and environmental results:** Results from water tests showed that the turbidity was > 5 Nephelometric Turbidity Units at the mini-water treatment plant and water tap in village 6 and free residual chlorine was high (> 0.5) compared with the acceptable range recommended by the Rwanda Standards Board for drinking water. The microbiological tests did not identify any coliform. Microbiological analysis of nine food and beverage samples taken at the community markets in both the old and new sites of the camp identified sorghum beverages (local beer) and local yoghurt were contaminated with yeast, aerobic flora, coliforms or Staphylococcus.

## Discussion

This study describes the epidemiology of Salmonella transmission in the Mahama refugee camp. These data were useful for evaluating and implementing the outbreak response strategy and ultimately contributed to the control of the outbreak. This study identified having a family member affected by typhoid fever in the last 3 months, poor awareness about how to prevent and control the disease, inconsistent hand washing after going to the latrine, consumption of food prepared at home and from the community market as risk factors for the disease. This proves our study hypothesis that transmission was not associated with consumption of contaminated water. Furthermore, the laboratory analysis of the water samples taken from the camp showed that the water was biologically safe for consumption, which further supports this theory. In addition, the epidemic curve of this outbreak illustrates a propagated outbreak, which is highly suggestive of person-to-person transmission, and confirms our finding of having a family member affected as one of the main risk factors for transmission. The association of increased risk with poor awareness of typhoid fever is not surprising, as lack of awareness is known to be associated with poor compliance with typhoid prevention and control practices [12]. The association between eating food prepared at home and in the community market with higher risk of disease transmission could be because of a number of factors. The laboratory analysis showed that the food and beverages sampled at the community market were contaminated. Furthermore, observations during the environmental assessment highlighted poor enforcement of food safety and hygiene in the community markets. Utensils such as cups were not properly cleaned and were being shared between multiple clients of the alcoholic beverage sellers, while house fly control was also a major challenge. Additional sampling and epidemiological analysis showed clustering of typhoid fever cases around the pit latrines. There was lack of adequate space in the camps, therefore many families were observed to be preparing and consuming their food next to open waste water trenches and pit latrines, which could increase the risk of food contamination. This is corroborated by previous studies in Zimbabwe and Indonesia, which showed that crowded living conditions and poor sanitation were associated with outbreaks of typhoid fever [13, 14].

Additionally, inconsistent washing of hands after using the latrine, which is another risk factor identified in this study, may have contributed to the association between eating food prepared at home and in the market and disease transmission. These findings are supported by studies conducted in Indonesia, Vietnam and India, where having close contact with cases and poor hand washing practices, with no use of soap, were risk factors for typhoid fever transmission [14, 15]. Furthermore, Bhunia et al [16] reported a strong association with eating food from a shop in which an infected patient was working and engaging in unsafe food handling practices and contamination. Our findings showed that females and children aged under 15 years of age were most at risk. This finding is consistent with results from studies conducted in Nigeria, Uganda, and Malaysia, which reported a high prevalence of typhoid infection among females and children in the same age group (< 15 years) [17,18]. As care-givers in African societies, women are known to be at increased risk of infection from infectious diseases. Observations during the environmental assessment highlighted young children were playing in and around open waste water trenches that are likely to be contaminated. Furthermore, overcrowded living conditions were observed in villages 10 and 11, which had the highest attack rates during this outbreak. Indirect environmental transmission may have also contributed to disease spread [19] and transmission of infection via house flies from human waste to food may also be a possibility in this environment [20]. This is consistent with the finding that typhoid fever cases were clustered around latrines, where houseflies are usually prevalent. The signs and symptoms such as fever, abdominal pain and headache that were reported in this study are consistent with the usual symptoms of typhoid fever. The long duration of time (on average 5.7 days) before seeking treatment, which was observed in this study, could be because of many factors. Lack of information about how the disease is prevented and controlled could have been responsible for this. The considerable proportion of people self-medicating (23.5%) could have delayed seeking treatment. Additionally, the long waiting time before receiving treatment at the only fully operational health center at the time of the study may have discouraged early attendance at the center. Teke et al [21] reported lack of good services as being the major motivator for self-medication in refugees in Durban, which further supports this hypothesis. Furthermore, delay in seeking health care was also reported by Srikantiah et al [22] during an investigation of typhoid fever in Uzbekistan.

**Study limitations:** The study was conducted 3 months after the typhoid fever outbreak and prevention and control interventions had already been introduced, therefore knowledge and attitudes regarding sanitation and hygiene practices may have changed. As a result, common risk factors associated with the disease may have been masked during our investigation. Information was retrospectively self-reported and provided by mothers or care givers for participants aged under 15 years, which could have introduced some recall bias into the study. These limitations were addressed through rigorous training of data collectors, pre-testing of the questionnaire and reformulation of questions, where necessary. There is a high proportion of asymptomatic illness associated with S. Typhi, therefore it is possible that some asymptomatic carriers may have been misclassified as controls. These would markedly reduce the observed association between cases and potential exposure because of the similarity between cases and controls. In addition, given the broad symptomatology associated with typhoid fever, which is similar to other viral enteritis illnesses, there could have been an overestimation of the true typhoid fever cases. To address these limitations, the ratio of controls to cases was increased from two to three.

## Conclusion

This study identified having a family member affected by typhoid fever, inconsistent hand washing, poor awareness about preventive measures and consumption of food prepared at home and from the community market as the main risk factors for typhoid fever in Mahama camp. In view of these findings, we proposed four main recommendations to facilitate timely containment of this and future outbreaks. First, hygiene promotion activities should be reinforced in the camp, particularly among those who are at highest risk of being infected such as those households accommodated near the latrines and those with previously infected people in the household. Furthermore, we recommend continued respect of the sanitation corridor between latrines and households during shelter planning in future. In addition, when planning relocation of refugees to semi-permanent houses, priority should be given to refugees accommodated in the crowded hangars in the old site, to reduce the overcrowded conditions. Second, food safety and hygiene regulations should be enforced in the community markets. This should be complemented with engagement of the market association members and market users to improve hygiene education. Furthermore, provision of more hand washing facilities, particularly in the community markets should be prioritized. Third, hygiene education for alcohol beverage sellers and food handlers in the market on potential diseases outbreaks associated with poor water, sanitation and hygiene should be increased. Finally, environmental interventions, particularly those related to sanitation, should be reinforced during critical times, such as the rainy season and periods of increased temperature, where an increase in the house fly population is possible [23].

### What is known about this topic

- The risk factors associated with typhoid fever are commonly known as contaminated water and food;
- The population in situations of mass displacement such as refugee camps is exposed to communicable diseases such as typhoid fever;
- Improved quantity and quality of drinking water in refugee camp, enhanced knowledge, attitude and practice towards prevention and control of poor hygiene related outbreaks among displaced people reduce the risk of water and foodborne disease outbreaks such as typhoid fever.

### What this study adds

- The transmission of typhoid fever is not only associated with the consumption of contaminated water and food, but inconsistent hand washing practices are also the drivers of the disease in a situation of refugee camp settings such as Mahama camp, highlighting the need of improvement of hygiene promotion activity with more emphasize on correct and consistent hand washing.
